# Clinical manifestations and immune markers of non-HIV-related CMV retinitis

**DOI:** 10.1186/s12879-024-09653-x

**Published:** 2024-08-06

**Authors:** Olga Passarin, Florence Hoogewoud, Oriol Manuel, Yan Guex-Crosier

**Affiliations:** 1 Department of Ophthalmology, University of Lausanne, Jules-Gonin Eye Hospital, Fondation Asile des Aveugles, avenue de France 15, Lausanne, 1002 Switzerland; 2 Cabinet ophtalmologique, Av. de la Gare 52, Lausanne, 1003 Switzerland; 3 Department of Infectiology, Centre Hospitalier Universitaire Vaudois, University of Lausanne, Lausanne, 1011 Switzerland

**Keywords:** CMV retinitis, Recurrence, Lymphocyte count, CD4 + lymphocytes, Immunoglobulins, Cytomegalovirus, Immunosuppression, Solid organ transplant, Infectious uveitis

## Abstract

**Background:**

Since the HIV epidemic in the 1980s, CMV retinitis has been mainly reported in this context. CMV retinitis in persons living with HIV is usually observed when CD4 + cells are below 50 cells/mm3. This study aims to describe the immune markers of non-HIV-related CMV retinitis as well as to describe its clinical manifestations and outcomes.

**Methods:**

Retrospective chart review of consecutive patients with CMV retinitis not related to HIV seen at the uveitis clinic of Jules Gonin Eye Hospital between 2000 and 2023. We reported the clinical manifestations and outcomes of the patients. We additionally assessed immune markers during CMV retinitis (leukocyte, lymphocyte, CD4 + cell and CD8 + cell counts as well as immunoglobulin levels).

**Results:**

Fifteen patients (22 eyes) were included. Underlying disease was hematologic malignancy in 9 patients, solid organ transplant in 3 patients, rheumatic disease in 2 patients and thymoma in one patient. The median time between the onset of underlying disease and the diagnosis of retinitis was 4.8 years. Lymphopenia was observed in 8/15 patients (mild = 3, moderate = 4, severe = 1), and low CD4 counts were observed in 9/12 patients, with less than 100 cells/mm3 in 4 patients. Hypogammaglobulinemia was detected in 7/11 patients. Retinitis was bilateral in 7/15 patients, and severe visual loss was frequent (5/19 eyes). Disease recurrence was seen in 7/13 patients at a median time of 6 months after initial diagnosis. No differences in immune markers were observed in patients with vs. without recurrence.

**Conclusion:**

CMV retinitis is a rare disorder that can affect patients suffering any kind of immunodeficiency. It is associated with a high visual morbidity despite adequate treatment. CD4 + cell counts are usually higher than those in HIV patients, but B-cell dysfunction is common.

## Background

Cytomegalovirus (CMV) is a double-stranded DNA virus from the Herpesviridae family. The seroprevalence of CMV infection in the population is estimated to be between 50 and 100%. [1] Retinitis is a sight-threatening condition and represents one of the most severe presentations of CMV infection. Since the HIV epidemic in the 1980s, CMV retinitis has been mainly reported in this context. CMV retinitis in persons living with HIV (PLWH) is usually observed when CD4 + cells are below 50 cells/mm3 and represents one of the most frequent opportunistic infections defining AIDS [2] . Even though most of the literature on CMV retinitis is HIV-related, CMV retinitis due to iatrogenic immunosuppression has been known since the forties [3]. The immune response to CMV infection and the mechanism of CMV reactivation remain poorly understood. Whereas the role of CD4 + and CD8 + T lymphocytes in the control of CMV infection has been largely demonstrated, the impact of the use of immunosuppressive drugs impairing humoral immunity on the incidence of CMV retinitis has been less reported [4].

This study aims to describe the clinical characteristics, immune markers and clinical outcomes of non-HIV-related CMV retinitis in a cohort of patients seen in our outpatient clinics over the last two decades.

## Methods

This is a retrospective study of consecutive patients treated for non-HIV-related CMV retinitis between 2000 and 2023 in the uveitis clinic of Jules-Gonin Eye Hospital in Lausanne, Switzerland. The study was conducted in compliance with the principles of the Declaration of Helsinki and was approved by the Institutional Review Board (CER-VD n° 2018–02161) for retrospective analysis between 2000 and 2022. For later inclusion all patient had signed the informed consent allowing data analysis for research purpose (CER-VD PB 2016 − 00868).

Written informed consent was obtained from all living patients, for deceased patient, the use of data was approved by the above-mentioned ethic committee.

CMV retinitis diagnosis was based on the SUN (Standardized Uveitis Nomenclature study group) classification criteria. [5] Briefly, it was suspected based on the presence of a retinal necrotic lesion with indistinct borders and small satellite lesions in the context of immunosuppression. It was confirmed either by a PCR on aqueous humor after anterior chamber tap or by the presence of additional clinical characteristics (wedge-shaped lesion, hemorrhagic retinitis and/or granular appearance) associated with a confirmed systemic CMV infection. All the included patient had a negative antigenic and serologic test for HIV infection.

Demographic data collection included age, sex, systemic disease, immunosuppressive therapy, delay of onset of CMV retinitis after introduction of immunosuppression (in autoimmune disease or after solid organ transplantation) or chemotherapy in hematologic malignancies, laterality of eye involvement, and type and duration of antiviral therapy. Clinical data collected included best corrected visual acuity (BCVA) using the decimal chart. The zonal distribution of the lesions was evaluated according to Holland et al. [6]^,^ where zone 1 refers to an area of 3000 μm (2 disc diameters) from the fovea or 1500 μm from the edge of the optic nerve head, zone 2 extends up to the equator and zone 3 is the area beyond the equator. The extent of the lesion was expressed in clock-hours of retinal involvement. The presence of complications (retinal detachment, ischemia, etc.) was also reported. Follow-up data were described in patients with a follow-up longer than 6 months and included final BCVA, number of recurrences, antiviral prophylaxis duration and, if applicable, time to death.

Laboratory data collected included the number of CMV copies measured by quantitative polymerase chain reaction (qPCR) in the peripheral blood, in the aqueous humor and in the cerebrospinal fluid when appropriate (CSF) (in one case). The total leucocyte count was reported as well as the lymphocyte, CD4+, CD8 + and CD19+ (B-lymphocytes) counts. Leukopenia was classified into mild (3 - < 4 G/L), moderate (< 3–2 G/L) and severe (< 2 G/L) forms according to an adapted classification from the National Cancer Institute Common Terminology Criteria for Adverse Events (NCI-CTAE). [7] Similarly, lymphopenia was graded into mild (500–1000 cells/mm^3^), moderate (200–499 cells/mm^3^) and severe (< 200 cells/mm^3^) forms. Gammaglobulin levels were also reported, and hypogammaglobulinemia was classified into severe (< 3.5 G/L) and moderate (3.5-5 G/L) forms [8].

Categorical variables were expressed as numbers (percentages). For continuous variables, the median and range were reported. The Wilcoxon-Mann‒Whitney test was used to compare the results. A *p* value < 0.05 was considered statistically significant.

## Results

Fifteen consecutive patients (22 eyes) were included in the study. This represents an incidence rate of 0.49% of uveitis cases in our tertiary referral center. The median age was 66 years (IQR 14). Seven patients were males (46.7%). Systemic diagnosis was hematologic malignancies in 9 patients (60%), solid organ transplant (SOT) in 3 patients (20%), rheumatic disease in 2 patients (systemic lupus erythematosus (SLE) and rheumatoid arthritis) (13.3%), and one patient presented with a thymoma and Good syndrome (Table 1)(6.7%).

**Table 1:** Demographic data, systemic diagnosis and treatment

Patient number	Age at the time of retinitis diagnosis (years), Gender	Systemic diagnosis	Type of immunosuppressive treatment at the time of retinitis diagnosis	Delay (months) between systemic diagnosis or solid graft and retinitis	CMV infection known before retinitis diagnosis	Other end-organ CMV disease	Donor (D) and Recipient (*R*) serostatus
1	67, M	Chronic Lymphatic Leukemia	Rituximab, cyclophosphamide, fludarabin	58	Absent	No	NA
2	17, M	Lymphoblastic Leukemia	Methotrexate, 6-thioguanin	52	Present	No	NA
3	66, M	Angioimmunoblastic T-Cell Lymphoma	Methotrexate, 6-thioguanin	13	Absent	No	NA
4	75, F	Follicular non-Hodgkin Lymphoma	Rituximab, bendamustine	111	Absent	No	NA
5	54, F	Follicular non-Hodgkin Lymphoma	Rituximab, bendamustine	26	Absent	Gastrointestinal Neurological	NA
6	52, F	T-Cell Prolymphocitic Leukemia	None	28	Present	Hepatic	NA
7	47, F	Acute lymphoblastic leukemia	Mercaptopurine, methotrexate	25	Present	No	NA
8	69, F	Lymphocytic variant hypereosinophilic syndrome	Methotrexate, Prednisone	280	Present	No	NA
9	67, F	Multiple myeloma	Teclistamab	131	Absent	No	NA
10	55, M	Thymoma with Good syndrome	None	121	Absent	No	NA
11	72, F	Rheumatoid arthritis	Baricitinib, methotrexate	573	Present	No	NA
12	64, F	Systemic lupus erythematosus	Mycophenolate mofetil, prednisone	304	Absent	No	NA
13	54, M	Cardiac transplantation	Tacrolimus, mycophenolate mofetil, prednisone	8	Absent	No	D-/R+
14	67, M	Cardiac transplantation	Cyclosporine, Mycophenolate mofetil, prednisone	4	Absent	No	D unknown, R+
15	73, M	Lung transplantation	Tacrolimus, mycophenolate mofetil, prednisone	97	Present	Gastrointestinal	D-/R+

The median time between systemic diagnosis or SOT and retinitis was very variable (4.8 years, IQR 8.4 years). Patients with autoimmune diseases presented the longest disease duration prior to the onset of retinitis (47.8 and 25.4 years, respectively) as opposed to heart transplant recipients who presented with CMV retinitis 4 and 8 months, respectively, after transplantation (Table 1).

CMV retinitis was bilateral in 7 patients (46.7%). The zonal distribution and extent of the lesions are depicted in Table 2. Initial BCVA was severely impaired (≤ 1/10 BCVA) in 4/22 eyes (18.2%).

**Table 2:** Ophthalmological characteristics and follow up data

Patient number	Follow-up (years)	Delay between retinitis diagnosis and death (years)	Laterality	Zonal distribution of the lesions and extent	Systemic treatment duration*	Adjuvant intra-vitreal injections (number)	Recurrence	Initial BCVA of affected eyes	Final BCVA	Ocular complications
1	8.8	8.8	bilateral	OD : Zone 3, 2 h OS : Zone 3, 2 h	valganciclovir po (8 months)	no	yes, at 8 months	OD 0.6 OS 0.6	OD 0.1 OS 0.5	OU Immune recovery uveitis with macular edema OD Retinal detachment
2	21.0	living	bilateral	OD : Zone 1, 2 h OS : Zone 1, 1 h	foscarnet iv (6 weeks), valganciclovir po (3 months)	no	no	OD 1.0 OS 0.6	OD 1.0 OS 1.0	None
3	0.4	0.4	unilateral	Zone 2, 4 h	valganciclovir po (1 month)°	no	yes, at 3 months	0.6	0.4	None
4	4.7	4.7	bilateral	OD: Zone 1, 8 h OS: Zone 1, 3 h	valganciclovir po (24 months)	yes [10]	no	OD 0.1 OS 0.5	OD HM OS 0.5	OD macular branch retinal artery occlusion
5	2.1	2.1	unilateral	Zone 1, 2 h	ganciclovir iv (10 days), valganciclovir po (17 months)	no	yes at 17 months	CF	No LP	Macular extension of retinitis
6	2.5	2.5	bilateral	OD: Zone 3, 1 h OS: Zone 1, 1 h	cidofovir iv(4 weeks), foscarnet iv (9 months)	yes [6]	yes at 10 months	OD 1.0 OS CF	OD 0.4 OS CF	OD Retinal detachment OS Macular branch retinal artery occlusion
7	1.9	living	bilateral	OD: Zone 2, 5 h OS: Zone 3, 1 h	valganciclovir po (7 months)	no	no	OD 1.0 OS 0.9	OD 0.9 OS 0.4	OU: macular edema due to immune recovery uveitis OS: glaucoma surgery
8	0.3	living	unilateral	Zone 2, 6 h	Valganciclovir po (ongoing)	no	NA	0.5	NA	None
9	0.3	living	bilateral	OD : Zone 1, 6 h OS : Zone 1, 3 h	Valganciclovir po (ongoing)	no	NA	OD: HM OS: 0.3	NA	Macular extension of the retinitis
10	1.4	1.6	bilateral	OD: Zone 2, 1 h OS: Zone 2, 2 h	valganciclovir po (5 months)	no	yes, at 5 months	OD 1.0 OS 0.8	OD 1.0 OS 0.8	none
11	0.5	0.5	unilateral	Zone 2, 3 h	valganciclovir po (7 months)	no	no	1.0	1.0	none
12	3.1	living	unilateral	Zone 2, 4 h	valganciclovir po (12 months)	no	no	0.5	0.25	Retinal detachment
13	1.3	living	unilateral	Zone 3, 3 h	valganciclovir po (4 months)	no	yes, at 5 months	0.6	0.16	Retinal ischemia due to occlusive vasculitis. Macular edema
14	1.2	living	unilateral	Zone 3, 4 h	valganciclovir po 3 weeks	no	no	1.0	0.8	Retinal detachment, cataract surgery
15	0.9	living	unilateral	Zone 3, 1 h	valganciclovir po (4 months)	no	yes, at 6 months	0.5	1.0	none

OD: right eye, OS: left eye, OU: both eyes, BCVA: best corrected visual acuity, CF: counting fingers, LP: light perception, HM hand movement, po: per os, iv: intraveinous, NA: not applicable because of a short duration of follow-up. *It refers to the treatment of the first episode of CMVR and includes the duration of the treatment at therapeutic and prophylactic doses. ° Treatment stopped early due to severe pancytopenia. NA follow up data were not applicable if the follow up was less than 6 months

CMV DNA in peripheral blood was present in 11/13 patients (84.6%). It was known prior to the diagnosis of CMV retinitis in 6/13 patients (46.2%), discovered at the time of CMV retinitis in 5/13 patients (38.5%) and remained negative in 2/13 patients (15.4%).

The main laboratory features at the time of diagnosis are depicted in Table 3. The median leucocyte count was 5 G/L (IQR 3.4), with severe leukopenia present in 2 patients (13.3%). The median lymphocyte count was 846 cells/mm3 (IQR 993), with only two patients presenting severe lymphopenia (13.3%), and the median CD4 count was 348 cell/m^3^ (IQR 407), with 4/12 patients with counts lower than 100 cell/mm^3^ (33.3%). A CD8 + count lower than 400 cell/mm^3^ was present in 6 patients (50%), with no data for 3 patients. The median gammaglobulin level was 5.43 G/L (IQR 6.25), with hypogammaglobulinemia present in 7 out of 11 patients (63.6%).

**Table 3:** Laboratory features at the time of CMV retinitis diagnosis

Patient number	Total Leucocyte count	Total Lymphocyte Count	CD4 + T Lymphocyte Count	CD8 + T Lymphocyte Count	NK Lymphocyte Count	B Lymphocyte Count	CD4/CD8 Ratio	Gamma-globulin level	CMV viral copy number in the aqueous humor	CMV viral copy number in the blood
Reference Value	4–10 G/L	1000–3380 cell/mm^3^	(490–1640) cell/mm^3^	(170–880) cell/mm^3^	(80–690) cell/mm^3^	(80–490) cell/mm^3^	1.3–2.4	7-14.5 G/L		Negative
1	5.7	2210	**350**	583	NA	NA	0.6	**2.63**	10,065	NA
2	**1.1**	**154**	< 154*	NA	NA	NA	NA	**4.59**	NA	2520
3	4.7	**846**	**382**	423	NA	**0**	0.9	NA	872	NA
4	5	1742	**110**	1699	NA	NA	0.06	**3.2**	NA	3800
5	**2.2**	**219**	**19**	173	NA	NA	0.1	**2.63**	NA	5’000’000
6	**1.8**	**389**	**9**	**55**	212	110	0.16	9.85	NA	8710
7	4.5	1400	**470**	596	NA	NA	0.79	**5.88**	13,000	20,170
8	**3.6**	**355**	**32**	194	64	**3**	0.16	9.87	NA	3’430’000
9	**2.2**	**500**	NA	NA	NA	NA	NA	NA	3’670’000	15,900
10	5.8	3459	550	2604	NA	NA	0.2	**4.03**	1038	450
11	7.8	1130	**347**	600	**48**	**0**	0.58	**5.43**	6900	400
12	5.3	1330	888	290	65	118	3.1	13.2	663	289,427
13	7.3	**500**	**87**	199	NA	NA	0.44	NA	600	Negative
14	9.7	**197**	< 197*	NA	NA	NA	NA	NA	NA	12,700
15	6.79	939	513	347	**26**	**44**	1.47	12.8	26,000	Negative

Abnormal values are presented in bold. Severely affected values are additionally underlined. NA: not available. * CD4 count unavailable but at least as low as the total lymphocyte count

Initial treatment was oral valganciclovir (900 mg bid adjusted to the renal function) in 12 cases (80%) or intravenous ganciclovir (5 mg/kg bid) in 1 case (6.7%). Patients 2 and 6 initially received alternative treatments (foscarnet, 60 mg/kg/day TID and/or 5 mg/kg/week cidofovir) because of a known systemic ganciclovir-resistant CMV infection (proven in patient 6 by a mutation N408K in the UL54 gene). Induction therapy was given until full scarring of the lesion for a median time of 30 days (range 21–90 days). It was followed by maintenance therapy of 7 months (range from 3 weeks in one patient presenting with pancytopenia to 24 months). Two patients received adjuvant intravitreal foscarnet injections (1.2 mg in 0.05 mL): patient number 4 because of pancytopenia and patient number 6 because of a known ganciclovir-resistant virus.

Two patients were excluded from the follow-up analysis (follow-up of less than 6 months). The median follow-up after diagnosis was 1.9 years (IQR 1.9) (Table 2). Seven patients (53.8%) died during the follow-up at a median time of 2.1 years (IQR 2.55) after retinitis diagnosis. Of the 7 patients, 2 (15.4%) died during the first year after the diagnosis. The number of deaths corresponded to a rate of 0.06 patients/year.

Recurrence occurred in 7 patients (53.8%) after a median time of 6 months after initiation of therapy (IQR 6). Three of those patients presented multiple recurrences (four recurrences for patient 6 and two recurrences for patients 10 and 13). The calculated recurrence rate was 24% recurrences per patient/year. Four patients were still under valganciclovir maintenance therapy (450 mg BID) at the time of recurrence. From the 7 patients with recurrence, four responded well to increased dosage of ganciclovir, one patient had a known resistant strain and was treated with cidofovir and two patients (number 5 and 10) were suspected to have to ganciclovir resistant virus because of progression of the disease under therapeutic dosage. Both responded well to alternative treatments (Foscavir and cidofovir respectively). The other three had stopped the antiviral treatment: 2 months earlier in 2 patients and one month earlier in the third. Of the 19 eyes with CMV retinitis, BCVA at last follow-up was severely impaired (≤ 1/10) and moderately impaired (2/10 − 5/10) in 4 (21.1%) and 7 (36.8%) eyes, respectively. Ocular complications included retinal detachment in 4 eyes (21.1%), retinal ischemia and/or vascular occlusion in 2 eyes (10.5%), macular edema due to immune recovery in 4 eyes (2 patients, 21.1%) and foveal extension of the retinitis in one case.

There were no statistically significant differences in lymphocyte and CD4 counts or globulin levels in patients with or without relapse (Table 4).

**Table 4:** Immune markers in patients with and without relapse of CMV retinitis

Variable	Patients with recurrence *N* = 7	Patient without recurrence *N* = 6	*P*-value
Total leucocyte count, G/L mean (SD) median (range)	4.9 (2.1) 5.7 (1.8–7.3)	5.6 (3.0) 5.2 (1.1–9.7)	0.943
Total lymphocyte count, cell/mm^3^ mean (SD) median (range)	1223 (1185) 846 (212–3459)	992 (663) 1230 (154–1742)	0.943
**CD 4 + count**,** cell/mm**^**3**^ **mean (SD)** ***median (range)***	273 (231) 350 (9-550)	361 (291) 408 (110–888)	0.508
**IgG levels**,** G/L** **mean (SD)** ***median (range)***	6.4 (4.7) 4.0 (2.6–12.8)	6.5 (3.9) 5.43 (3.2–13.2)	0.529

## Discussion

This study presents the data of 15 patients (22 eyes) with non-HIV-related CMV retinitis. Supporting the results of other series, we found hematological malignancies to be the most frequent systemic diagnoses associated with CMV retinitis [9]. Nevertheless, CMV retinitis can also be found in long-lasting rheumatismal diseases or after solid organ transplant [10].

The presentation of CMV retinitis in our series was often severe. We found zone 1 involvement in 36.4% of the eyes and an extension of over one-quarter of the retina in 45.5% of the eyes. Initial visual acuity below 5/10 was present in 40.9%. These results correspond to the current literature, which reports rates of zone 1 involvement in non-HIV-related CMV retinitis to vary between 33% and 61% [9–12]. We found that CMV retinitis in hematological malignancies more frequently presented bilateral involvement and a zone 1 extension of the lesions, in line with the results of a previous study [13].

The immune status of our patients was different from those found in HIV-related CMV retinitis. Whereas CMV retinitis is rarely seen in PLWH with CD4 + counts over 50 cell/mm^3^ and virtually never in patients with CD4 + counts over 100 cell/mm^3^, we found that only four patients (33.3%) had CD4 + counts under 100 cell/mm^3^. We found one series reporting CD4 + counts under 100 cell/mm^3^ in non-HIV-related CMV retinitis in 48% of their patients without details on the lymphocyte counts or immunoglobulin levels [12]. The total lymphocyte and CD4 + cell counts observed in our cohort are in line with previous publications of SOT and HSCT recipients with systemic CMV disease. After heart transplantation, a median lymphocyte count of 380 cell/mm^3^ was found in patients who developed early CMV disease compared to 840 cell/mm^3^ in patients without CMV disease [14]. Likewise, lymphopenia was identified as an independent risk factor for CMV disease after renal transplantation [15]. However, there are incomplete data on the level of immune parameters specifically for SOT recipients with CMV retinitis.

We also observed that immunoglobulin levels were reduced in 63.6% of the patients who were tested, a rate that was much higher than that usually found after SOT (45%) or after HSCT (32.5%). [16, 17] Hypogammaglobulinemia has been associated with an increased risk of CMV disease in these populations, [16, 18, 19]. In PLWH, gammaglobuline levels are frequently increased and hypogammaglobulinemia is a rare finding which is not known to be associated with CMV disease [20–23]. Given the uncontrolled nature of our study, we cannot exclude hypogammaglobulinemia as a marker of severity of the underlying disease rather than a risk factor for CMV retinitis. Therefore, the role of the substitution of immunoglobulins in these patients also remains under debate [24, 25]. 

Testing for CMV DNA in the blood by qPCR is a useful tool for the monitoring and detection of systemic CMV disease. The combination of a positive PCR test in the blood and visual symptoms should trigger a rapid ophthalmological evaluation. However, as shown for CMV colitis, our series demonstrated that 15.4% of our patients did not have CMV viremia, highlighting the necessity to maintain a high clinical suspicion for CMV retinitis in immunosuppressed patients even in the absence of signs of non-ophthalmic organ involvement of CMV disease. Early diagnosis remains a key feature for good visual outcome [26, 27]. Monitoring the CMV specific immune response might be a promising alternative to predict the risk of CMV disease, although the performance of these assays for predicting CMV retinitis has not been largely investigated [28, 29].

The prognosis of CMV retinitis is serious, with one-third of patients with severe visual loss at the final visit and half of the patients with recurrence in our series. These rates match most of the published series in non-HIV-related CMV retinitis [9, 10, 12]. One exception is a prospectively followed cohort of patients with hematological malignancies and CMV retinitis reporting a lower recurrence rate (16.7%), which might be explained by the inclusion of asymptomatic, less severe CMV retinitis (48% of the patients). [27] The mortality rate was also high (overall 53.8%, 15.4% at one year) but was lower than the rates reported in the literature (30.3% at one year in HSCT with CMV retinitis). [27]

In our study, due to the modest sample size, we were not able to correlate the use of a specific immunosuppressive drug and the risk for a worse outcome of CMV retinitis. It is, however, worth noting that all but three patients were taking two or more immunosuppressant drugs described as risk factors for CMV disease in the literature [30, 31]. 

Recurrence occurred in 42.8% of the cases under prophylactic treatment (3/7 cases). It is unknown whether these recurrences are related to a dose reduction of the prophylactic treatment due to systemic toxicity or to the development of drug-resistant strains. In any case, it highlights the need for alternative prophylactic treatments. New drugs have recently been developed for CMV prophylaxis and therapy, including letermovir and maribavir. Because maribavir does not cross the hematoencephalic barrier, it cannot be used for the treatment of CMV retinitis. Letermovir is currently used for CMV prophylaxis in HSCT recipients. Given the potential toxicity of long-term prophylaxis with valganciclovir, this drug can be an option for secondary prophylaxis to avoid relapse of CMV retinitis.

The main limitation of this study is the restricted number of patients and the retrospective nature of the study. Even though all patients had an iatrogenic immunosuppression, the different nature of the underlying diseases and treatments do not allow a direct comparison. Follow-up were also variable as well as the duration of prophylactic treatments. A prospective investigation of gammaglobuline levels, lymphocyte number and function should be perform to confirm our findings.

In conclusion, CMV retinitis is a rare disease affecting immunosuppressed patients. It is associated with a high mortality rate and a high morbidity rate. Compared with HIV-related CMV retinitis, phenotyping of lymphocytes revealed less frequently reduced CD4 + and CD8 + counts. Hypogammaglobulinemia was very frequent, but its significance requires further investigation.

## Data Availability

The datasets used and/or analysed during the current study are available from the corresponding author on reasonable request.
